# Homozygous R396H mutation of the *RAG1* gene in a Saudi infant with Omenn’s syndrome: a case report

**DOI:** 10.4076/1757-1626-2-8391

**Published:** 2009-07-30

**Authors:** Mohammed Al Balwi, Sulaiman Al Ajaji, Ibrahim Al Abdulkareem, Ali Hajeer

**Affiliations:** 1 King Saud bin Abdulaziz University for Health Sciences, College of MedicineRiyadh, 11426Saudi Arabia; 2 Department of Pathology & Laboratory MedicineKing Abdulaziz Medical City Riyadh, 11426Saudi Arabia; 3 Department of Pediatric Medicine, King Abdulaziz Medical CityRiyadh, 11426Saudi Arabia; 4 King Abdullah International Medical Research Center, Molecular Biology, King Abdulaziz Medical CityRiyadh, 11426Saudi Arabia

## Abstract

**Introduction:**

The V(D)J rearrangement of B and T cell lymphocytes during the recombination process, which is essential for the development of normal immune system function, depends critically on the presence of the recombination activating enzymes, *RAG1* and *RAG2*. Mutations in *RAG1* or *RAG2* can lead to a spectrum of disorders, ranging from typical B-T-severe combined immunodeficiency to Omenn’s syndrome.

**Case presentation:**

A two-month-old Saudi baby girl presented with fever, respiratory distress due to bronchiolitis, exfoliative erythroderma and a family history of childhood death within the first few months of life in two of her sisters who had had a similar clinical presentation to her own. Immunological work-up revealed an absence of circulating B lymphocytes, whereas various numbers of activated T lymphocytes were present in the peripheral blood and in the skin.

**Conclusion:**

In this case, mutation analysis of the recombination activating genes *RAG1* or *RAG2* revealed a homozygous missense (c.1299G>A) mutation in the *RAG1* gene. This is the first report in the literature linking a homozygous R396H mutation in the *RAG1* gene with presentation of Omenn’s syndrome.

## Introduction

Omenn’s syndrome (MIM 603554) is an autosomal recessive form of severe combined immunodeficiency (SCID) characterized by early-onset generalized exfoliative erythroderma, desquamation, alopecia, chronic diarrhea, failure to thrive, lymphadenopathy, hepatosplenomegaly and elevated IgE levels. In early infancy, patients with SCID develop potentially life-threatening recurrent infections that may be caused by common bacterial, fungal, or viral infections.

Laboratory findings reveal a characteristic hypereosinophilia and hypogammaglobulinemia. However, IgE serum levels are increased paradoxically in the absence of detectable B lymphocytes in peripheral blood and in skin and lymph node tissues [1,2].

Both B and T cell lymphocytes undergo the recombination process of V(D)J rearrangement that is essential for the development of normal immune function, and the recombination activating enzymes *RAG1* and *RAG2* play a crucial role in this recombination [3]. Molecular genetic studies have been focused on elucidating the molecular basis of the B-T- subgroup of SCID that is associated with Omenn’s syndrome.

Mutation analysis studies involving SCID and Omenn’s syndrome have identified mutations in *RAG1* and *RAG2* [1] that may show differing clinical presentations depending on the type of mutation. When mutations in the recombinase genes *RAG-1* and *RAG-2* have been explored, homozygous and heterozygous mutations have been found. In contrast to T cell negative (T^−^), B cell negative (B^−^), and natural killer cell positive (NKC^+^) SCID, in which *RAG-1* and *RAG-2* mutations affect the active core of the gene, homozygous mutations affecting the active core have not been observed in Omenn’s syndrome. Approximately half of the mutations are missense mutations, while the remainder are nonsense, deletion, frameshift, duplication, and splice mutations[1,4].

**Figure 1.**
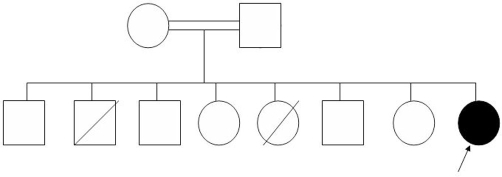
Pedigree of the Saudi family with Omenn’s syndrome. Blackened symbol indicate proband. Diagonal lines across symbols indicate deceased individuals.

Here, we describe a Saudi female infant who was the 8^th^ child of first-degree relative parents (Figure 1). Family history was positive for two early infantile deaths with clinical manifestations consistent with Omenn’s syndrome, as shown in this patient. The patient was shown to be a homozygote for mutations in the *RAG1* gene.

## Case presentation

Our case is that of a two-month-old Saudi female (Figure 1). The family has a history of two early infantile deaths from undiagnosed recurrent chest infections associated with generalized skin erythema. Pregnancy and delivery were uncomplicated. Birth weight was 3500 g. At the age of 2 weeks, the infant presented with generalized scaly erythroderma associated with persistent fever and later developed pneumonia. There was no lymphadenopathy and no hepatosplenomegaly. Subsequently, she developed protracted diarrhea and failure to thrive. The patient had elevated numbers of circulating white blood cells and low hemoglobin (Hb). Flow cytometry showed a critically low (absent) B-cell count with a predominance of T cells (82%) that showed high memory (CD45RO) and activation (CD25) expression. There were normal levels of natural killer cells with normal expression of gamma/delta TCR cells, but no detectable CD19+ or CD20+ B-cells. Both CD4 and CD8 T cells were significantly suppressed; only 21% of CD4 and 33% of CD8 were activated in response to phytohemaglutinine (PHA). Humoral immunity was also abnormal, with elevated serum IgE levels. The nature of the T cells and the remarkable absence of the circulating B cells raised the possibility of a defect in immunoglobulin gene recombination. The histopathological finding of the skin biopsy revealed atypical lymphoid cells that are occasionally noted in the dermal infiltrate. The immunostaining showed that most of the lymphoid cells infiltrating the dermis were of T cell type. The staining of S-100 (polyclonal) was negative in the target cell, suggesting an absence of normal follicles. *RAG* mutation analysis of our patient revealed a homozygous missense mutation (c.1299G>A) in the *RAG1* gene leading to replacement of the normal Arginine (R) amino acid to Histidine (H) at the 396 codon position (R396H) of the resultant normal protein.

This is the first report in the literature showing a homozygous R396H mutation in the *RAG1* gene presenting itself with features of Omenn’s syndrome. Our patient presented with some of the typical clinical manifestations of Omenn’s syndrome that are characterized by a generalized early-onset of exfoliative erythroderma, persistent infection, alopecia, elevated IgE level and elevated white blood count due to lymphocytosis. Skin biopsy showed autologous T-cell infiltration and the absence of a germinal center, which is consistent with histopathological findings in Omenn’s syndrome [5].

The patient had no B cells, and the majority of her WBCs were T cells that had a very high expression of the CD45RO memory marker and IL-2R (CD25). Both CD4 and CD8 T cells showed significantly reduced capacity to respond to PHA stimulation. Villa et al. (2001) and Corneo et al. (2001) described patients who showed a nearly complete absence of B cells, reduced number of T cells, and normal NK.

The *RAG* mutation analysis in our patient revealed a homozygous missense mutation (c.1299G>A) in the *RAG1* gene, which led to the replacement of the normal Arginine amino acid to Histidine at the 396 codon position of the resultant normal protein. This mutation has been previously reported in association with Omenn’s syndrome in which this missense mutation (c.1299G>A) was detectable in a patient who was heterozygous for the allele; the mutation was associated with a protein-truncating mutation [4,6].

The location of this missense mutation (i.e., R396H) is within the *RAG1* homeodomain region, which is responsible for the recognition of and binding to the nonamer motif of the recombination signal sequence (RSS) [6]. Biochemical analysis performed on classic Omenn’s syndrome cases that are harboring a one-allele missense mutation in their *RAG1* gene maintained partial V(D)J recombination activity, which accounted for the generation of detectable numbers of activated, anergic, oligoclonal T-lymphocytes [6]. In our case, the lack of circulating B cells, together with oligoclonal expansion of T cells, raised the possibility of *RAG* deficiency despite the clinical presentation. We suspect that the presence of a homozygous R396H mutation in our patient likely destroyed the function of the *RAG1* protein and hence impaired the recombination activity that may be associated directly or indirectly with impairment in B cell immunity. The result of this may be an increased occurrence of life-threatening infections, and, without proper treatment, the condition may lead to death within the first year of life. This explains the death of our patient's two sisters who presented with symptoms consistent with Omenn’s syndrome during their few months of life.

## Conclusion

This is the first report in the literature showing a homozygous R396H mutation in the *RAG1* gene being linked to features of Omenn’s syndrome. Consanguineous marriages increase the risk of autosomal recessive disorders. Therefore, choosing to undergo premarital screening, prenatal diagnosis and genetic counseling should be an important consideration for family future planning.

## Abbreviations

NKC: natural killer cell
SCID: severe combined immunodeficiency
